# Rapid screening of pyogenic *Staphylococcus aureus* for confirmation of genus and species, methicillin resistance and virulence factors by using two novel multiplex PCR

**DOI:** 10.12669/pjms.335.13487

**Published:** 2017

**Authors:** Abdul Haque, Asma Haque, Muhammad Saeed, Aysha Azhar, Samreen Rasool, Sidra Shan, Beenish Ehsan, Zohaib Nisar

**Affiliations:** 1 Abdul Haque, PhD. Postgraduate Research Laboratory, The University of Faisalabad, Faisalabad, Pakistan; 2 Asma Haque, PhD. Department of Bioinformatics and Biotechnology, GC University, Faisalabad, Pakistan; 3 Muhammad Saeed, PhD. Postgraduate Research Laboratory, The University of Faisalabad, Faisalabad, Pakistan; 4 Aysha Azhar, PhD. Postgraduate Research Laboratory, The University of Faisalabad, Faisalabad, Pakistan; 5 Samreen Rasool, PhD. Postgraduate Research Laboratory, The University of Faisalabad, Faisalabad, Pakistan; 6 Sidra Shan, M.Phil Postgraduate Research Laboratory, The University of Faisalabad, Faisalabad, Pakistan; 7 Beenish Ehsan, M.Sc. Department of Bioinformatics and Biotechnology, GC University, Faisalabad, Pakistan; 8 Zohaib Nisar, M.Sc. Department of Bioinformatics and Biotechnology, GC University, Faisalabad, Pakistan

**Keywords:** Pyogenic staph, MRSA, Virulence factors, Multiplex PCR

## Abstract

**Objectives:**

Emergence of methicillin resistant *Staphylococcus aureus* (MRSA) is a major medical problem of current era. These bacteria are resistant to most drugs and rapid diagnosis can provide a clear guideline to clinicians. They possess specific virulence factors and relevant information can be very useful. We designed this study to develop multiplex PCRs to provide rapid information.

**Methods:**

We studied 60 *Staphylococcus aureus* isolates and detected methicillin resistance by cefoxitin sensitivity and targeting of *mecA* gene. After initial studies with uniplex PCRs we optimized two multiplex PCRs with highly reproducible results. The first multiplex PCR was developed to confirm genus, species and methicillin resistance simultaneously, and the second multiplex PCR was for screening of virulence factors.

**Results:**

We found 38.33% isolates as methicillin resistant. α -toxin, the major cytotoxic factor, was detected in 40% whereas β-hemolysin was found in 25% cases. Panton Valentine leucocidin was detected in 8.33% and toxic shock syndrome toxin in5% cases. The results of uniplex and multiplex PCRs were highly compatible.

**Conclusions:**

These two multiplex PCRs when run simultaneously can provide vital information about methicillin resistance and virulence status of the isolate within a few hours as compared to several days needed by routine procedures.

## INTRODUCTION

*Staphylococcus aureus* is a major human pathogen causing a wide range of diseases.^1^,^2^ The methicillin resistant *Staphylococcus aureus* (MRSA) bacteria are referred to as “superbugs”. They are now commonly found in both nosocomial and community acquired infections.^3^

*Staphylococcus aureus* usually cause pyogenic diseases and they are armed with specific virulence factors. These include alpha-hemolysin, beta hemolysin, and Penton Valentine leucocidin (PVL), and toxic shock syndrome toxin (TST).^4^

PVL production has been preferentially linked to furuncles, cutaneous abscesses, and severe necrotic skin infections. TST, as its name suggests causes toxic shock syndrome which is a life threatening condition and hemolysins lyze the red blood cells.^4^,^5^ Information about the presence of these virulence factors can provide vital clues to the clinician and epidemiologists.

In this study, pyogenic *Staphylococcus aureus* were analyzed for methicillin resistance and virulence factors. The data obtained was used to develop two multiplex PCRs for rapid confirmation of genus and species, to detect methicillin resistance with its genotypic variations, and to assess presence/absence of common virulence factors of pyogenic *Staphylococcus aureus*.

## METHODS

### Sample collection

Pus samples (no = 102) from wounds, ear, and skin were collected from outpatient department and indoor patients of Madinah Teaching Hospital (600 bed), The University of Faisalabad, Faisalabad, Pakistan. The samples were aseptically collected on sterile swabs and transferred to laboratory within one hour and processed immediately.

### Cultivation and bacterial isolation

The swabs were inoculated on blood agar and nutrient agar plates overnight at 37°C. The suspected *Staphylococcal* colonies were sub-cultured on the same media for obtaining pure cultures and to record colonial morphology. Probable *Staphylococcal* colonies were found in 72 cases. They were processed for biochemical and molecular identification.

### Coagulase test

The isolates suspected as *Staphylococcus* were processed for Gram-staining and observed under light microscope. The coagulase test was performed on glass slides to detect coagulase positive *Staphylococci*.^6^

### Phenotypic detection of Methicillin resistance

By definition, *mecA* gene is present in all methicillin-resistant *Staphylococcus aureus* (MRSA) isolates. Cefoxitin disk diffusion test is the most accurate phenotypic test to check the presence of the *mecA* gene in *Staphylococcus aureus* because cefoxitin is a more potent inducer of *mecA* expression than other agents and the test results are relatively easy to interpret.^7^ The test isolate is inoculated heavily on Mueller Hinton agar with 2% sodium chloride under standardized conditions and a cefoxitin disk (30 mcg) is applied. A zone of growth inhibition around the cefoxitin disk of ≥22 mm rules out MRSA; a zone size <22 mm indicates that the *mecA* gene is present and the isolate should be reported as MRSA.

### DNA extraction and purification

DNA extraction and purification was done according to a reported protocol.^8^

### Monoplex PCR for virulence factors

Virulence factors associated with pyogenic *Staphylococcus aureus* were searched for by targeting relevant genes according to reported protocols. The targeted virulence factors included Panton Valentine leucocidin (PVL), toxic shock syndrome toxin, and alpha and beta hemolysins. References are given in Table-I.

**Table-I:** Primers used in this study.

*No.*	*Targeted Genes/Virulence Factors*	*Sequences*	*Product Size (bps)*	*Reference*
1	16s RNA	AAC TCT GTT ATT AGG GAA GAA CA CCA CCT TCC TCC GGT TTG TCA CC	756	10
2	nuc	GCGATTGATGGTGATACGGT AGCCAAGCCTTGACGAACTAAAGC	280	10
3	mecA	GGT CCC ATT AAC TCT GAA G (mA1) ATC GAT GGT AAA GGT TGG C (mA2) AGT TCT GCA GTA CCG GAT TTG C (mA3)	mA1-mA3 1046 bp mA2-mA3 540 bps	9
4	PVL	ATC ATT AGG TAA AAT GTC TGG ACA TGA TCCA GCA TCA AGT GTA TTG GAT AGC AAA AGC	433	10
5	Alpha hemolysin	GAA GTC TGG TGA AAA CCC TGA TGA ATC CTG TCG CTA ATG CC	704	11
6	Beta hemolysin	CAA TAG TGC CAA AGC CGA AT TCC AGC ACC ACA ACG AGA AT	496	11
7	TST	ACC CCT GTT CCC TTA TCA TC TTT TCA GTA TTT GTA ACG CC	326	12

### Multiplex PCR for genus, species and methicillin resistance

A multiplex PCR was developed to confirm genus, species and methicillin resistance simultaneously. The 16s rRNA and *nuc* genes were targeted to confirm *Staphylococci* and *Staphylococcus aureus* producing amplifications products of 756 bp and 280 bp respectively (Table-I). Three primers were used to detect methicillin resistance as shown in Table-I.^9^ These primers were developed to detect normal *mecA* gene as well as its mutated forms. Two bands of 1046 bps and 540 bps indicates unmutated *mecA* gene whereas absence of either of these bands indicate two forms of mutated gene.

Hundred µL of reaction mixture was made up of 7 µL dNTPs, 5 µL of 10x PCR buffer, 3 µL MgCl_2_, 2 µL of each primer, 1.5 µLTaq polymerase (Fermentas, USA), 10 µL template DNA and 10 mM Tris buffer (pH 8.3) to make the volume. The thermal cycler conditions for first step were 1 cycle at 94°C for 10 minutes. It was followed by 35 cycles each of heating at 94°C for 60 sec; at 45°C for 60 sec; and at 72°C for 75 sec. Final step was heating at 72°C for 10 minutes. The amplification products were analyzed by electrophoresis at 100V using 2.5% agarose gel.

### Multiplex PCR for virulence factors

For easy screening of virulence factors of pyogenic *Staphylococcus aureus*, we developed another multiplex PCR. This multiplex PCR included 16s rRNA gene as internal control and genes for Panton Valentine leucocidin (PVL), toxic shock syndrome toxin, and alpha and beta hemolysins.

Hundred µL of reaction mixture was made up of 7 µL dNTPs, 5 µL of 10x PCR buffer, 3 µL MgCl_2_, 2 µL of each primer, 2.0 µLTaq polymerase (Fermentas, USA), 10 µL template DNA and 10 mMTris buffer (pH 8.3) to make the volume. The thermal cycler conditions for first step were 1 cycle at 94°C for 10 minutes. It was followed by 35 cycles each of heating at 94°C for 60 sec; at 48°C for 60 sec; and at 72°C for 60 sec. Final step was heating at 72°C for 10 minutes. The amplification products were analyzed by electrophoresis at 100V using 2.5% agarose gel.

## RESULTS

### Genus and species confirmation

In all the 60 isolates the 16s rRNA gene that confirms the genus and the *nuc* genes that confirm *Staphylococcus aureus* were detected.

### Methicillin resistance

Out of 60 *Staphylococcus aureus* isolates, 23 (38.33%) were methicillin resistant phenotypically. Twenty (86.95%) among them were genotypically confirmed. There was one isolate which was genotypically positive but phenotypically negative. The methicillin resistance results were almost similar in indoor (12; 37.5%) and outdoor (11; 39.28%) patients.

Among the 20 genotypically positive isolates, 11 (55.00%) showed both 540 and 1046 bp bands, whereas 7 (35.00%) showed only the 540 bp band. Two (10.00%) isolates showed 1046 bp band only.

### Distribution of virulence factors

Among these 60 *Staphylococcus aureus* isolates, 5(8.33%) were positive for PV leucocidin, 3(5.00%) for TST, 24 (40.00%) for α-hemolysin and 15 (25.00%) for β-hemolysin. Distribution among indoor and outdoor patients is given in Table-II.

**Table-II:** Distribution of virulence factors among pyogenic Staphylococcus aureus.

	*PV leucocidin*	*Toxic shock syndrome toxin*	*α- hemolysin*	*β- hemolysin*
Indoor patients (32)	3 (9.37%)	1(3.12%)	10 (31.25%)	8 (25.00%)
Outdoor patients (28)	2 (7.14%)	2(7.14%)	14 (50.00%)	7 (25.00%)
Total (60)	5 (8.33%)	3 (5.00%)	24 (40.00%)	15 (25.00%)

### Multiplex PCR 1 for genus and species confirmation and methicillin resistance

**Fig.1:**
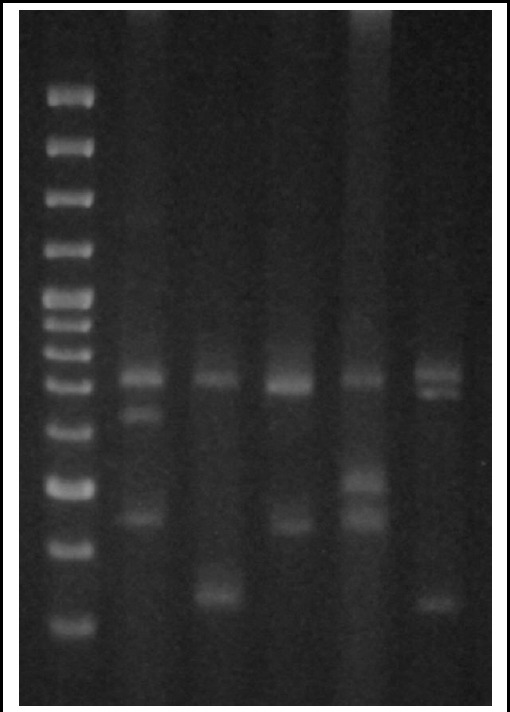
Multiplex PCR for genus, species and methicillin resistance detection of Staphylococcus aureus ***Lane 1:*** MW marker SM 0321 showing bands of 3000, 2000, 1500, 1200, 1000, 900, 800, 700, 600, 500, 400, 300, 200, 100 bps respectively. ***Lane 2-6:*** All isolates showing genus specific 16s rRNA gene (756 bps) which was used as internal control. ***Lane 2 and 4:*** Isolates showing PV gene (433 bps). ***Lane 3:*** Isolate showing *tst* gene (326 bps): Isolate showing β-hemolysin (496 bps) and PV genes. ***Lane 6:*** Isolate showing α-hemolysin (704 bps) gene and *tst* gene.

The above mentioned results were obtained by uniplex PCR. They were highly reproducible with the developed multiplex PCR. Representative results are shown in Fig.1.

### Multiplex PCR 2 for virulence factors

**Fig.2:**
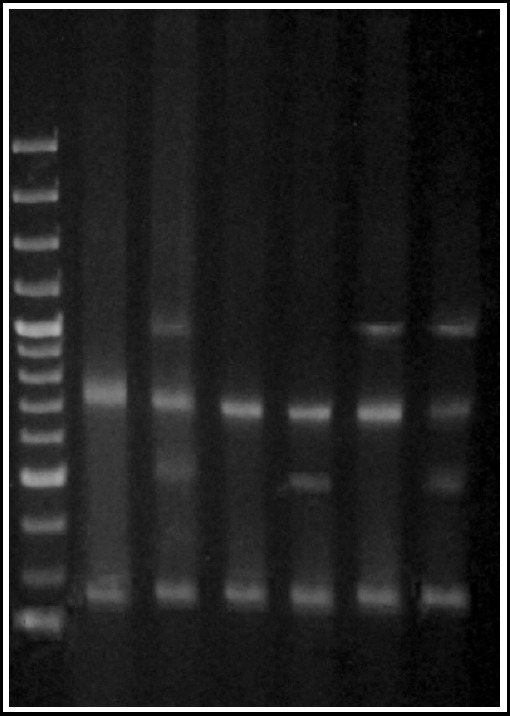
Multiplex PCR for detection of genes for virulence factors alpha hemolysin, beta hemolysin, Panton Valentine leukocidin and toxic shock syndrome toxin of Staphylococcus aureus. ***Lane 1:*** MW marker SM 0321 showing bands of 3000, 2000, 1500, 1200, 1000, 900, 800, 700, 600, 500, 400, 300, 200, 100 bps respectively. ***Lanes 2 & 4:*** Isolates showing amplification products for 16s rRNA(756 bps) and *nuc* (240 bps) genes only. ***Lane 5:*** Isolate showing an additional amplification product of 540 bps indicating presence of a mutated *mecA* gene. ***Lane 7:*** Isolate showing an additional amplification product of 1046 bps indicating presence of a mutated *mecA* gene. ***Lane 3 & 7:*** Isolates showing both amplification products of *mecA* gene indicating unmutated gene.

The results by multiplex PCR were highly reproducible when compared with results of uniplex PCR. Representative results are shown in Fig.2.

## DISCUSSION

*Staphylococci* are among the most important human pathogens. Approximately 30% of the human population is colonized with *Staphylococcus aureus*.^13^ Although some strains cause food poisoning, the majority cause pyogenic infections which can be life threatening.

The targeting of 16s rRNA genes is generally considered to be the most dependable method for bacterial identification.^14^ We targeted these genes for identification of *Staphylococcus* genus by using previously reported primers.^10^

*Staphylococcus aureus* strains produce an extracellular thermostable nuclease (thermonuclease [TNase]) with a frequency similar to that at which they produce coagulase. The TNase protein is well characterized, and its gene, the *nuc* gene, has been cloned and sequenced. It is a reliable indicator that the organism is *Staphylococcus aureus*.^15^ We chose to target this gene by using previously reported primers.^10^

Methicillin resistance has emerged rapidly in *Staphylococcus aureus*. In USA, MRSA (methicillin resistant *Staphylococcus aureus*) was first detected in hospital infections in 1960s and in community associated infections in 1981.^16^ Rapid identification of MRSA is of great significance as it makes the treatment decisions easier and definitive. Methicillin resistance rules out use of a number of drugs.

Phenotypic method using cefoxitin is approved by CLSIand is considered as most dependable.^7^ We found that 23 (38.33%) of our isolates were methicillin resistant. The results were almost similar in indoor (12; 37.5%) and outdoor (11; 39.28%) patients. Our results show lesser incidence than that stated in another report from Pakistan who reported 51% isolates as methicillin resistant.^17^ However, they are in agreement with a large scale study (8987 isolates) conducted in USA.^18^ They reported figures of 31.8%.

We used the primers that can detect not only the *mecA* gene (the most common methicillin resistant gene), but also its mutated forms.^9^ We were able to detect unmutated *mecA* gene and its mutated forms in 20 (90.91%) isolates among 22 phenotypically positive isolates. Conversely one isolate was genotypically positive but phenotypically negative. In 11 (55.00%) isolates, the unmutated *mecA* gene was detected, whereas in 9 (45.00%) the *mecA* gene was mutated at two different positions [7 (35.00%); and 2 (10.00%)]. The absence of *mecA* gene in some phenotypically positive isolates may be because some other genes such as *mecR1* and *mecI* may also be responsible for methicillin resistance.^9^

α-toxin (α–hemolysin) is the major cytotoxic agent of *Staphylococcus aureus*. Systemic release of α-toxin causes septic shock. In humans, platelets and monocytes are particularly sensitive to α-toxin. We were able to detect its gene in 24 (40.00%) cases. β-hemolysin was found in 15 (25.00%) cases. A previous report has mentioned detection rates of 34.88% and 42.60% for α- and β-hemolysins respectively.^11^ It has also been reported that not all clones express alpha toxin and β-hemolysin.^18^

PVL specifically acts on polymorphonuclear leukocytes. It forms pores in the affected membrane. We were able to detect PVL in 5 (8.33%) cases. The results are similar to a previous study which reported 9.92% occurrence if cases of furunculosis and pneumonia are excluded.^19^ Similarly our results for toxic shock syndrome toxin (3; 5.00%) are comparable (3.40%) with a previous report.^20^

We were able to detect the genes for methicillin resistance and virulence factors with the similar efficacy as reported in literature individually and after multiplexing.

The multiplex PCR 1 (for genus/species confirmation and methicillin resistance) can be used for rapid identification and methicillin resistance determination to provide critical guidance to clinicians about treatment options as MRSA are resistant to a large number of drugs. Phenotypic procedures take almost 48 hours whereas this multiplex PCR can provide information within a few hours. The multiplex PCR 2 (for virulence factors) can give quick information about presence of major virulence factors. This information can be especially useful from epidemiological point of view.
